# Effect of Age on Blood Rheology in Sickle Cell Anaemia and Sickle Cell Haemoglobin C Disease: A Cross-Sectional Study

**DOI:** 10.1371/journal.pone.0158182

**Published:** 2016-06-29

**Authors:** Céline Renoux, Marc Romana, Philippe Joly, Séverine Ferdinand, Camille Faes, Nathalie Lemonne, Sarah Skinner, Nathalie Garnier, Maryse Etienne-Julan, Yves Bertrand, Marie Petras, Giovanna Cannas, Lydia Divialle-Doumdo, Elie Nader, Daniela Cuzzubbo, Yann Lamarre, Alexandra Gauthier, Xavier Waltz, Kamila Kebaili, Cyril Martin, Arnaud Hot, Marie-Dominique Hardy-Dessources, Vincent Pialoux, Philippe Connes

**Affiliations:** 1 Unité de Pathologie Moléculaire du Globule Rouge, Laboratoire de Biochimie et de Biologie Moléculaire, Hôpital Edouard Herriot, Hospices Civils de Lyon, Lyon, France; 2 Laboratoire Interuniversitaire de Biologie de la Motricité, Equipe “Vascular Biology and Red Blood Cell”, Université Claude Bernard Lyon 1, Lyon, France; 3 UMR Inserm U1134, Université des Antilles et de la Guyane, Hôpital de Pointe-à-Pitre, Hôpital Ricou, 97159 Pointe-à-Pitre Cedex, Guadeloupe, France; 4 Unité Transversale de la Drépanocytose, Hôpital de Pointe-à-Pitre, Hôpital Ricou, 97159 Pointe-à-Pitre Cedex, Guadeloupe, France; 5 Institut d’Hématologie et d’Oncologie Pédiatrique, Hospices Civils de Lyon, Lyon, France; 6 Service de Médecine Interne, Hôpital Edouard Herriot, Hospices Civils de Lyon, Lyon, France; 7 Laboratoire d’Excellence du Globule Rouge (LABEX GR-Ex), PRES Sorbonne, Paris, France; 8 Institut Universitaire de France (IUF), Paris, France; Loyola University Chicago, UNITED STATES

## Abstract

**Objectives:**

Blood rheology plays a key role in the pathophysiology of sickle cell anaemia (SS) and sickle cell haemoglobin C disease (SC), but its evolution over the lifespan is unknown.

**Materials and Methods:**

Blood viscosity, red blood cell (RBC) deformability and aggregation, foetal haemoglobin (HbF) and haematocrit were measured in 114 healthy individuals (AA), 267 SS (161 children + 106 adults) and 138 SC (74 children + 64 adults) patients.

**Results:**

Our results showed that 1) RBC deformability is at its maximal value during the early years of life in SS and SC populations, mainly because HbF level is also at its peak, 2) during childhood and adulthood, hydroxycarbamide treatment, HbF level and gender modulated RBC deformability in SS patients, independently of age, 3) blood viscosity is higher in older SS and SC patients compared to younger ones and 4) haematocrit decreases as SS patients age.

**Conclusion:**

The hemorheological changes detected in older patients could play a role in the progressive development of several chronic disorders in sickle cell disease, whose prevalence increases with age. Retarding these age-related haemorheological impairments, by using suitable drugs, may minimize the risks of vaso-occlusive events and chronic disorders.

## Introduction

Sickle cell disease (SCD) is a severe monogenic hereditary haemoglobin disorder characterized by chronic haemolytic anaemia and the occurrence of frequent, painful vaso-occlusive events. In addition, SCD patients experience progressive chronic organ damage and dysfunction with ageing.

The pathophysiology of SCD is complex and involves several factors such as impaired vascular reactivity, inflammation, oxidative stress, abnormal vascular adhesion and coagulation, and altered blood rheology [1,2,3,4,5,6]. Impaired blood rheology plays a key role in both acute and chronic complications in sickle cell anaemia (SS) and sickle cell haemoglobin C disease (SC; [3]). Decreased red blood cell (RBC) deformability is associated with leg ulcers [7], priapism [8] and glomerulopathy [9] in SS and with retinopathy in SC [10], while blood hyper-viscosity increases the risk of acute painful vaso-occlusive crises in SS [11,12] and otologic disorders in SC [10].

The evolution of several haematological indices, as well as foetal haemoglobin (HbF) level (a strong modulator of clinical severity in SS), have been well described in SCD populations from birth to adulthood and during ageing [13,14,15]. It has previously been shown that blood rheology may be influenced by age in the general population with blood viscosity and RBC aggregation increasing, and RBC deformability decreasing, with age [16]. However, the impact of age on the blood rheological characteristics of SCD patients has never been investigated until now. In the present study, we compared several blood rheological parameters between two large cohorts of SS and SC patients who were regularly examined in Lyon (France) and Pointe-à-Pitre (Guadeloupe, French West Indies), stratified according to age categories ranging from birth to more than 60 years of age.

## Design and Methods

### Patients

Four hundred and five SCD patients (165 from Lyon (France), and 240 from Pointe à Pitre (Guadeloupe, French West Indies)) were included in this study: 267 SS (161 children + 106 adults) and 138 SC (74 children + 64 adults). Diagnosis of SS and SC was made by isoelectrofocusing (Multiphor II^™^ System, GE HEALTH CARE, Buck, UK), citrate agar electrophoresis, and cation-exchange high performance liquid chromatography (VARIANT^™^, Bio-Rad Laboratories, Hercules, CA, USA). SS status was confirmed by DNA studies [17]. All patients were in steady-state condition at the time of the study: no blood transfusion in the previous three months and absence of acute episodes (infection, vaso-occlusive crises, acute chest syndrome, stroke, priapism) for at least two months before inclusion in the study. A control group (AA) from the same ethnic origin as the SCD patients was composed of 114 healthy individuals (48 males and 66 females) between the ages of 15 and 79 years old. All individuals were informed of the purpose and the procedures of the study, and gave their written consent. The study was conducted in accordance with the guidelines set by the Declaration of Helsinki and was approved by the Regional Ethics Committees “CPP Sud/Ouest Outre Mer III, Bordeaux, France” (registration numbers: 2010-A00244-35 and 2009-A00211-56) and the “Hospices Civils de Lyon—CPP Est” Ethics committee (L14-127), for Pointe-à-Pitre (Guadeloupe) and Lyon (France), respectively.

### Haemorheological parameters

Blood viscosity was measured after complete oxygenation of the blood, at native haematocrit (Hct), at 25°C and at a shear rate of 225 s^−1^ using a cone/plate viscometer (Brookfield DVII+ with CPE40 spindle, Brookfield Engineering Labs, Natick, MA) [18]. Hct was determined by microcentrifugation as recommended [18].

RBC deformability was determined at 37°C at 3 and 30 Pascals (Pa) by laser diffraction analysis (ecktacytometry), using the Laser-assisted Optical Rotational Cell Analyzer (LORRCA MaxSis, RR Mechatronics, Hoorn, The Netherlands). The system has been described elsewhere in detail [18]. Briefly, 25 μl of prepared blood suspension was mixed with 5 ml polyvinylpyrrolidone (PVP; viscosity = 30 cP) and sheared into a Couette system made of glass. The diffraction pattern was analysed by the computer and an elongation index was calculated. An increase of the elongation index indicates greater RBC deformability [18]. We strictly followed the recent methodological recommendations for RBC deformability measurement in SCD by ektacytometry [19].

RBC aggregation properties were determined at 37°C by laser backscatter method, using the LORRCA MaxSis (RR Mechatronics, Hoorn, The Netherlands), after adjustment of the Hct to 40% with autologous plasma [18,20]. Blood was inserted into the Couette system of the LORRCA and the RBC aggregates strength was determined using a re-iteration procedure [20]: 7 separate pre-defined shear rates between 7.5 s^-1^ and 800 s^-1^ were applied on the RBC suspension, with or without alternating disaggregation shear rate, to locate the minimal shear rate needed to prevent RBC aggregation.

### Statistical analysis

Age categories were constituted as follows: 0–3, 3–5, 5–10, 10–15, 15–20 and then by 10-year bands. The number of individuals per age categories was: 0–3 (25 SS, 6 SC), 3–5 (15 SS, 6 SC), 5–10 (50 SS, 20 SC), 10–15 (56 SS, 34 SC), 15–20 (31 SS, 14 SC, 10 AA), 20–30 (27 SS, 20 SC, 11 AA), 30–40 (34 SS, 19 SC, 18 AA), 40–50 (15 SS, 12 SC, 23 AA), 50–60 (14 SS, 7 SC, 19 AA) and 60–70 (5 SC, 33 AA) yrs old. RBC deformability and blood viscosity values for healthy individuals younger than 15 yrs old were recorded from previous reports using the same devices as those used in the present study [21,22,23]. Analysis of variances with post-hoc Tukey test was used to compare the different parameters between the 3 populations across the different age categories. The distribution of gender and hydroxycarbamide treatment in the different age categories was evaluated by a chi-square test. Independent student t test was used to test the effects of gender and hydroxycarbamide on the biological parameters in SC and SS patients. Pearson’s test was used to test for the presence of correlations between biological parameters. Multivariate linear regression models were performed to identify which covariates independently associated with the changes in hemorheological parameters with ageing. The significance level was defined as p < 0.05. Data are displayed as means ± SD. Statistical analyses were conducted using SPSS software (version 20, IBM SPSS Statistics, Chicago, IL, USA).

## Results

### Effects of age

**Fig 1:**
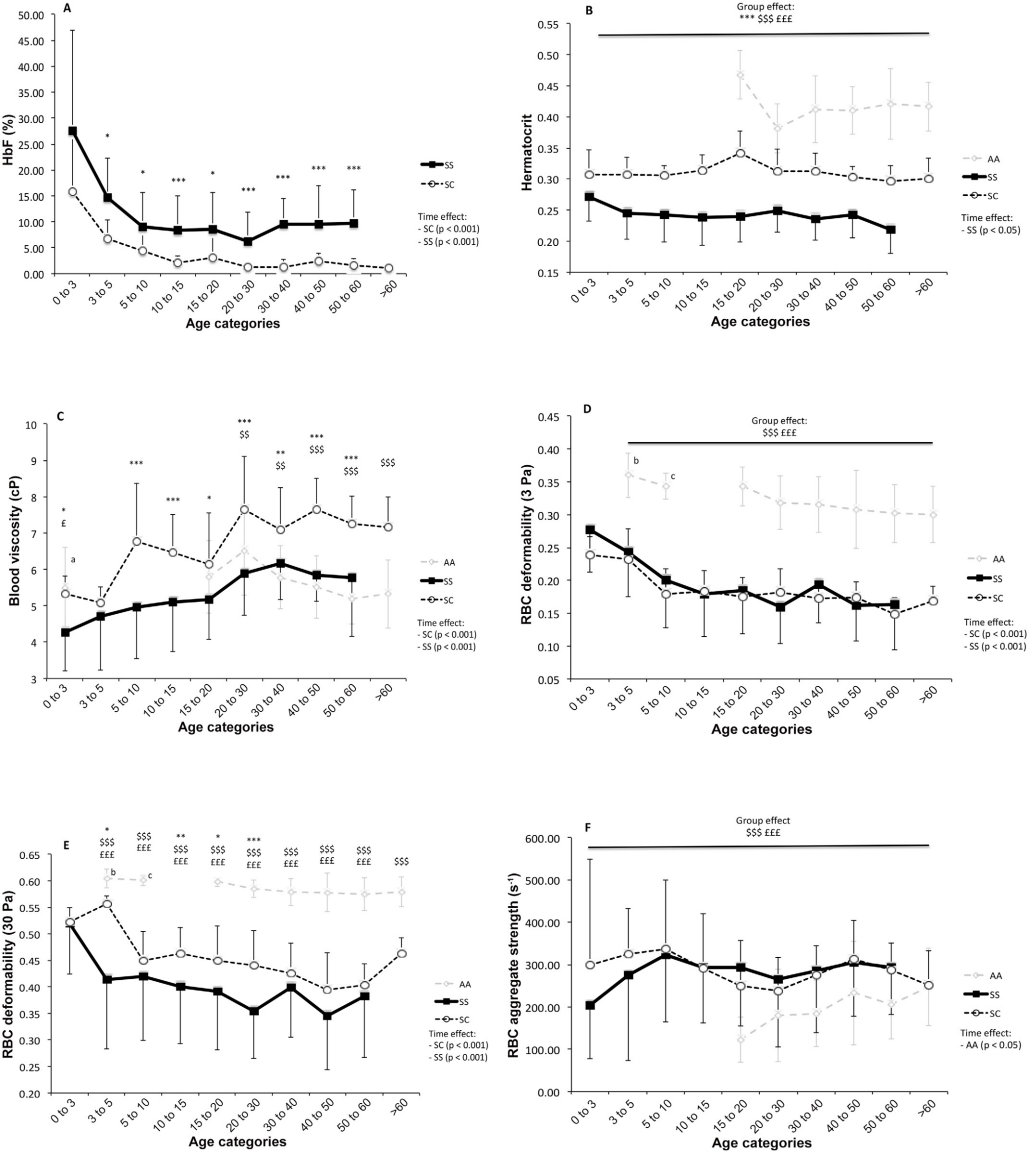
1A) Comparisons of foetal haemoglobin (HbF) between SS and SC patients at different age categories; 1B) Comparisons of haematocrit between controls (AA), SS and SC patients at different age categories; 1C) Comparisons of blood viscosity between AA, SS and SC patients at different age categories. ^a^Blood viscosity values from Mackintosh *et al* [22]; 1D) Comparisons of RBC deformability measured at 3 Pa between AA, SS and SC patients at different age categories. ^bc^RBC deformability values from Gurses *et al* [21] and Tancer-Elci *et al* [23]; 1E) Comparisons of RBC deformability measured at 30 Pa between AA, SS and SC patients at different age categories. ^bc^RBC deformability values from Gurses *et al* [21] and Tancer-Elci *et al* [23]; 1F) Comparisons of RBC aggregates strength between AA, SS and SC patients at different age categories. Difference between SS and SC (*p < 0.05; **p < 0.01; ***p < 0.001), SS and AA (^£^p < 0.05; ^£££^p < 0.001), SC and AA (^$$^p < 0.01; ^$$$^p < 0.001).

Fig 1A shows the HbF values for the different age categories in SS and SC patients. HbF was not significantly different between SS and SC in the 0–3 yrs old band, however HbF was systematically higher in SS than in SC for the older age categories (p < 0.05 to 0.001). For both groups (SS and SC), HbF values were maximal between 0 and 3 yrs old, and then were lower in the older age groups in SS (p < 0.001) and SC (p < 0.001). HbF values did not differ between the different age categories after 10 and 15 yrs old in SS and SC individuals, respectively. Hct (Fig 1B) was higher in AA than in SS and SC at all time points (p < 0.001), and higher in SC than in SS at all time points (p < 0.001). No difference in Hct was observed between the different age categories in SC or AA. In contrast, we observed an age category group effect for Hct in SS (p < 0.05) and Hct was significantly lower at 50–60 yrs old compared to 0–3 yrs old (p < 0.001).

Blood viscosity was lower in SS compared to AA and SC within the 0–3 yrs old age group (Fig 1C, p < 0.05). In SS (p < 0.001) and SC (p < 0.001), blood viscosity increased between each age category until 30–40 (SS) and 20–30 (SC) yrs old, at which point it stabilized at values higher than those measured in the younger categories. Blood viscosity of AA did not differ between the different age bands. Blood viscosity of AA and SS were not different within each age category that was tested, other than the 0–3 yrs old category. In contrast, blood viscosity was higher in SC than SS for all age categories from 5–10 to 50–60 yrs old (p < 0.05 to 0.001), and higher than AA for all age categories from 20–30 to > 60 yrs old (p < 0.01 to 0.001).

At 3 Pa, RBC deformability was higher in AA than in SS and SC, for all age categories considered (Fig 1D, p < 0.001). No differences were observed between SS and SC at this shear stress. RBC deformability at 3 Pa did not differ between age categories tested in AA. In contrast, we observed lower RBC deformability in the older compared to the younger age categories in SS and SC (p < 0.001), and more specifically when comparing the 0–3 with the 3–5 and 5–10 yrs bands (p < 0.05 to 0.001). Nevertheless, RBC deformability was also significantly lower in the late age category tested in SS compared to the 5–10 yrs band (p < 0.05). In the SC population, we noted a difference between the 5–10 yrs band and the 50–60 yrs band (p < 0.05) but not with the oldest category tested (i.e. > 60 yrs old).

At 30 Pa, RBC deformability was still lower in SS and SC compared to AA for all age categories (Fig 1E, p < 0.001). On the whole, we also observed a lower RBC deformability in SS than in SC patients (group effect: p < 0.001). However, when examined in more detail, the difference in RBC deformability between SS and SC patients reached statistical significance for the age categories 3–5, 10–15, 15–20 and 20–30 yrs old (p < 0.05 to 0.001). While RBC deformability at 30 Pa did not differ between the age categories tested in AA, we observed an effect of age categories in SS and SC (p < 0.001). As for RBC deformability determined at 3 Pa, RBC deformability measured at 30 Pa was higher at 0–3 yrs old compared to 3–5 yrs old in SS (p < 0.01) and 5–10 yrs old in SC (p < 0.01). Nevertheless, we also observed lower RBC deformability in SS patients from the 20–30 and 40–50 yrs old bands compared to patients from the 3–5 and 5–10 yrs old categories (p < 0.05). In SC, we observed lower RBC deformability in the 40–50 and 50–60 yrs old categories compared to the 5–10 yrs old band (p < 0.05).

In SS, HbF significantly correlated with RBC deformability at 3 (r = 0.54, p < 0.001) and 30 Pa (r = 0.46, p < 0.001) and with Hct (r = 0.43, p < 0.001). In addition Hct and RBC deformability were positively correlated (r = 0.54, p < 0.001 for the two shear stresses). In SC, HbF positively correlated with RBC deformability at 3 (r = 0.36, p < 0.001) and 30 Pa (r = 0.30, p < 0.01). Hct and RBC deformability at 30 Pa were also significantly correlated (r = 0.21, p < 0.05).

RBC aggregate strength did not differ between SS and SC but was systematically higher in the two SCD groups compared to AA at any time point (Fig 1F, p < 0.001). No difference between the different age categories was observed for SS and SC patients but there was a main effect of age category for RBC aggregates strength in AA (p < 0.05).

### Effects of gender

The distribution of gender was not different between the age categories in the SS (χ^2^ = 11.92; p = 0.20) and SC populations (χ^2^ = 15.67; p = 0.08). Table 1 summarizes the different biological parameters in males and females in the two SCD populations. SS females displayed higher HbF (p < 0.001), Hct (p < 0.05) and RBC deformability (p < 0.01 and p < 0.001 at 3 and 30 Pa, respectively) than SS males. There was no difference between the proportion of males and females undergoing hydroxycarbamide treatment (χ^2^ = 0.143; p = 0.71). Removal of patients under hydroxycarbamide treatment did not change the results (data not shown) and SS females still displayed higher HbF (p < 0.001), Hct (p < 0.05) and RBC deformability (p < 0.001 at 3 and 30 Pa). No gender difference was observed in SC patients.

**Table 1:** Effects of gender on haemorheological parameters in SS and SC patients.

	SS		SC	
	Males (n = 122)	Females (n = 145)	Males (n = 67)	Females (n = 71)
HbF (%)	7.5 ± 6.4	13.2 ± 11.8***	2.7 ± 3.1	3.1 ± 5.1
Hydroxycarbamide treatment (%)	25.6	23.6	0	0
Haematocrit	0.23 ± 0.05	0.25 ± 0.04*	0.32 ± 0.03	0.31 ± 0.02
Blood viscosity (cP)	5.15 ± 1.32	5.34 ± 1.37	6.64 ± 1.40	6.94 ± 1.30
RBC deformability (3 Pa, a.u.)	0.18 ± 0.08	0.21 ± 0.06**	0.18 ± 0.03	0.18 ± 0.04
RBC deformability (30 Pa, a.u.)	0.38 ± 0.12	0.43 ± 0.10***	0.44 ± 0.06	0.45 ± 0.07
RBC aggregates strength (s^-1^)	303 ± 155	274 ± 136	298 ± 143	288 ± 108

Means ± SD. HbF, foetal haemoglobin. Significant difference between Males and Females:

*p < 0.05

**p < 0.01

***p < 0.001

### Effects of hydroxycarbamide

Since HbF decreased significantly until 10 yrs of age in SS subjects, the comparison of biological parameters between SS patients treated by hydroxycarbamide and those who were not was performed for individuals older than 10 yrs old (Table 2). The proportion of patients undergoing hydroxycarbamide treatment did not differ between the different age categories analysed (i.e. from 10–15 to 50–60 yrs old; χ^2^ = 2.52, p = 0.77). HbF (p < 0.05), RBC deformability at 3 and 30 Pa (p < 0.01) were higher and blood viscosity (p < 0.001) was lower in SS hydroxycarbamide-treated patients compared to those who were not treated. Inclusion of children between the ages of 5 and 10 yrs old did not affect the results (data not shown).

**Table 2:** Effects of hydroxycarbamide on haemorheological parameters in SS patients older than 10 yrs old.

	SS after 10 yrs old	
	Hydroxycarbamide + (n = 49)	Hydroxycarbamide − (n = 128)
HbF (%)	10.3 ± 7.2	7.8 ± 5.9*
Haematocrit	0.24 ± 0.06	0.23 ± 0.04
Blood viscosity (cP)	4.91 ± 1.07	5.77 ± 1.26***
RBC deformability (3 Pa, a.u.)	0.20 ± 0.06	0.17 ± 0.06**
RBC deformability (30 Pa, a.u.)	0.42 ± 0.10	0.37 ± 0.10**
RBC aggregates strength (s^-1^)	287 ± 157	289 ± 130

Means ± SD. HbF, foetal haemoglobin. Significant difference:

*p < 0.05

**p < 0.01

***p < 0.001

### Multivariate analyses

Multivariate linear regression models including age, gender, HbF and hydroxycarbamide treatment were performed to test the independent determinants of Hct, RBC deformability and blood viscosity in SS patients. For Hct, the model was statistically significant (R^2^ = 0.203, df = 4, p < 0.001) and both age (beta = -0.124; 95% CI -0.001 to 0.000, p < 0.05) and HbF (beta = 0.401; 95% CI 0.001 to 0.002, p < 0.001) were independently associated. For RBC deformability measured at 3 Pa, the model was statistically significant (R^2^ = 0.346, df = 4, p < 0.001): age (beta = -0.196; 95% CI -0.001 to 0.000, p < 0.01), HbF (beta = 0.461; 95% CI 0.002 to 0.004, p < 0.001) and hydroxycarbamide treatment (beta = -0.118; 95% CI -0.038 to -0.001, p < 0.05) were independently associated, while gender was marginally associated (beta = 0.101; 95% CI -0.002 to 0.031, p = 0.089). At 30 Pa, the model was still significant (R^2^ = 0.265, df = 4, p < 0.001) and the 4 factors tested were independently associated with RBC deformability: age (beta = -0.15; 95% CI -0.002 to 0.000, p < 0.05), HbF (beta = 0.373; 95% CI -0.003 to 0.006, p < 0.001), hydroxycarbamide treatment (beta = -0.134; 95% CI -0.068 to -0.004, p < 0.05) and gender (beta = 0.150; 95% CI 0.006 to 0.064, p < 0.05). For blood viscosity, the model was statistically significant (R^2^ = 0.153, df = 4, p < 0.001) but only age (beta = 0.391; 95% CI 0.162 to 0.328, p < 0.001) remained independently associated.

Since HbF stabilized after 10 yrs of age in SS patients, the same multivariate analyses were performed after removing patients younger than 10 yrs old. For both Hct and blood viscosity, the results were similar to those obtained previously (i.e., including patients younger than 10 yrs old; data not shown). For RBC deformability measured at 3 Pa, the model was statistically significant (R^2^ = 0.274, df = 4, p < 0.001) but age was not independently associated. Instead, HbF (beta = 0.391; 95% CI 0.002 to 0.004, p < 0.001) and hydroxycarbamide treatment (beta = -0.149; 95% CI -0.040 to -0.001, p < 0.05) remained independently associated with RBC deformability, and gender was still marginally associated (beta = 0.142; 95% CI -0.002 to 0.036, p = 0.078). At 30 Pa, the model was still significant (R^2^ = 0.208, df = 4, p < 0.001) and the same results for RBC deformability at 3 Pa were observed: HbF (beta = 0.334; 95% CI 0.003 to 0.008, p < 0.001) and hydroxycarbamide treatment (beta = -0.122; 95% CI -0.061 to 0.004, p < 0.05) were independently associated, and gender was marginally associated (beta = 0.143; 95% CI -0.004 to 0.063, p = 0.087).

None of the SC patients was under hydroxycarbamide medication. Moreover, no effect of gender was observed in the SC population. Indeed, multivariate linear regression models including age and HbF were only performed to test the independent determinants of RBC deformability and blood viscosity in SC patients. The models tested for RBC deformability at both 3 (R^2^ = 0.189, df = 2, p < 0.001) and 30 Pa (R^2^ = 0.177, df = 2, p < 0.001) were significant, and both age (3 Pa: beta = -0.263; 95% CI -0.001 to 0.000, p < 0.01; 30 Pa: beta = -0.314; 95% CI -0.002 to 0.000, p < 0.01) and HbF (3 Pa: beta = 0.264; 95% CI 0.001 to 0.004, p < 0.01; 30 Pa: beta = 0.200; 95% CI 0.001 to 0.006, p < 0.05) were independently associated with RBC deformability. For blood viscosity, the model was significant (R^2^ = 0.142, df = 2, p < 0.01) and only age (beta = 0.341; 95% CI 0.011 to 0.044, p < 0.01) was independently associated.

When multivariate analyses were performed after removal of SC patients younger than 10 yrs old, the model including age and HbF to predict RBC deformability at 3 Pa was not significant. In contrast, the model to predict RBC deformability at 30 Pa was significant (R^2^ = 0.071, df = 2, p < 0.05) and only age (beta = -0.261; 95% CI -0.002 to 0.000, p < 0.05) was independently associated. The multivariate model focusing on blood viscosity remained significant (R^2^ = 0.072, df = 2, p < 0.05) with age (beta = 0.222; 95% CI 0.000 to 0.036, p < 0.05) independently associated.

Finally, since blood viscosity may be affected by Hct and RBC rheological properties [3], another multivariate analysis was performed, depending on the shear rate used, to identify which covariates (RBC deformability, RBC aggregate strength and Hct) were independently associated with blood viscosity in SS and SC. The model including these 3 covariates was significant for both groups (R^2^ = 0.12, df = 3, p < 0.001 and R^2^ = 0.26, df = 3, p < 0.001 in SS and SC, respectively) and both RBC deformability (beta = -0.28; 95% CI -5.26 to -1.29, p < 0.01 and beta = -0.51; 95% CI -14.38 to -7.24; p < 0.001 in SS and SC, respectively) and Hct (beta = 0.33; 95% CI 4.88 to 13.63; p < 0.001 and beta = 0.23; 95%CI 2.70 to 18.87; p < 0.01 in SS and SC respectively) remained independently associated with blood viscosity.

## Discussion

This report is the largest cross-sectional study to date that focuses on blood rheological characteristics of patients with sickle cell anaemia and sickle cell haemoglobin C disease from birth to 50–60 yrs old. Our main findings were that 1) RBC deformability peaks during the early years of life in SS and SC populations, mainly because HbF level is also at its peak, 2) during childhood and adulthood, hydroxycarbamide treatment, HbF level and gender modulate RBC deformability in SS patients, 3) blood viscosity is higher in older SS and SC patients than in younger ones and 4) haematocrit is affected by age category in SS patients.

HbF is the predominant haemoglobin from early gestation until 2 months after birth in individuals without haemoglobinopathy. In SS, the replacement of HbF by abnormal haemoglobin (i.e., HbS) is delayed and HbF levels usually remain elevated compared to healthy individuals [24], with higher HbF levels in SS females than in SS males [25] as detected in our study. The switch from HbF to abnormal haemoglobin in the early years in both SS and SC patients is usually concomitant with the first clinical expressions of the disease [24,26]. Our findings demonstrated that the decrease of HbF within the first age categories is also accompanied by a decrease in RBC deformability. After 10 yrs old, age was no longer a predictor of RBC deformability in SS patients, instead this hemorheological parameter was primarily affected by the level of HbF, gender, and hydroxycarbamide treatment. Of note, even after removal of patients under hydroxycarbamide medication, HbF level remains independently associated with RBC deformability in SS patients (data not shown). Therapies promoting the synthesis of HbF and the production of F-cells are of particular interest in decreasing the clinical severity of the complications experienced by SS patients [27]. Our findings showing that SS patients treated with hydroxycarbamide had higher HbF levels than those who were not treated are in agreement with previous studies [27,28]. The increased HbF levels caused by hydroxycarbamide treatment are the root cause of the increase in RBC deformability found in treated versus untreated patients, underlining the beneficial haemorheological effects of this drug, which ultimately improve tissue perfusion and decrease the risk of vaso-occlusive events [29].

Blood viscosity is a key determinant of peripheral vascular resistance. In SS, blood hyper-viscosity has been found to predict the frequency of vaso-occlusive crises because vascular reactivity is completely blunted and cannot compensate for any elevation in blood viscosity [3,11,12,30]. In SS patients, the lower levels of Hct observed in the oldest age category would theoretically result in lower blood viscosity during this period of life. However, we observed higher blood viscosity in the oldest age categories in SS, as well as in SC. In multivariate analyses, age remained the only factor independently associated with blood viscosity. This higher blood viscosity observed in older patients could play a role in the progressive development of chronic disorders such as osteonecrosis and retinopathy, whose prevalence increases with age in both SS and SC patients [10,31]. The reasons for the higher blood viscosity in older SS and SC patients compared to younger ones are unknown, but the results from the multivariate analyses also suggest that the lower RBC deformability observed in older patients is, at least partly, responsible for the high blood viscosity.

Previous studies reported RBC aggregation abnormalities in SCD compared to controls [3,32]. The present work confirms in a large group of SS and SC patients that pre-formed SCD RBC aggregates are stickier and more robust than those in AA controls. While RBC aggregate strength differed between the different age categories in AA, none of the factors tested in this study affected this parameter in SS and SC patients. The role of abnormal RBC aggregation in SCD pathophysiology has been poorly studied, but Lamarre *et al* [9] found increased RBC aggregate strength in SS patients with glomerulopathy compared to those without. In another study, Lamarre *et al* [30] also found increased RBC aggregate robustness in SC patients who had previously developed acute chest syndrome compared to those who had never exhibited this complication. Elevated RBC aggregate strength is thought to increase flow resistance at the entry of capillaries as RBC aggregates need to be completely dispersed before they can enter and negotiate small capillaries [33].

In conclusion, to the best of our knowledge, our study is the first to describe the haemorheological profile of large groups of SS and SC patients at different ages. Delaying age-related haemorheological impairments, via the use of drugs such as hydroxycarbamide, may help minimize the risks of vaso-occlusive events and chronic disorders.
